# Intraoral clinical examinations of pediatric patients with anticipatory anxiety and situational fear facilitated by therapy dog assistance: A pilot RCT

**DOI:** 10.1002/cre2.679

**Published:** 2022-10-19

**Authors:** Anne M. Gussgard, Kerstin Carlstedt, Malin Meirik

**Affiliations:** ^1^ Department of Clinical Dentistry UiT The Arctic University of Norway Tromso Norway; ^2^ The Uppsala University Hospital, Sweden, Child, and Adolescent Psychiatric Services Uppsala Sweden

**Keywords:** dental anxiety, patient compliance, random allocation, therapy animals

## Abstract

**Objective:**

To evaluate whether the presence of a certified therapy dog specially trained for working in a dental setting may facilitate dental care of anxious pediatric patients.

**Methods:**

The Norwegian Regional Committee for Medical and Health Research Ethics approved a randomized cross‐over trial with a study sample of *n* = 16 children aged between 6 and 12 years. The trial was registered on clinicaltrials.gov. Pediatric patients referred to specialist care at the Public Dental Service Competence Center of Northern Norway (TkNN) because of anxiety were invited to partake in the trial. Study participants met twice for an intraoral examination by a specialist pediatric dentist. Per random allocation, a therapy dog team was present in the clinic operatory during the clinical examination on the first or the second visit. The primary outcome was the assessment of patient compliance during the intraoral examination (yes/no). Secondary outcomes were measurements of child satisfaction and anxiety using the CFSS‐DS scale (Dental subscale of Children's Fear Survey Schedule) completed by a parent/guardian. Supplementary outcomes were salivary cortisol level, heart rate variability, and skin conductance.

**Results:**

Ten boys and six girls (mean age 8.5) were recruited. All completed both clinical visits and demonstrated full compliance while undergoing a dental examination. All study participants and guardians reported great satisfaction. The salivary cortisol level reduction during the clinical examination on the first visit decreased by 30% in the presence of the therapy dog and 20% without, while the decrease during the clinical examination on the second visit was 29% in the presence of the therapy dog and 3% without. Within the limitations of the experimental setup, the electrophysiological measurements were unreliable in the current study population.

**Conclusion:**

Dog‐assisted therapy in a dental care setting appears to have a positive effect on children with dental anxiety or children that avoid dental care.

## 1 INTRODUCTION

Many children show signs and symptoms of anticipatory anxiety and situational fear while receiving oral health care. Prevalence estimates range between 10% and 30%, depending on the study population and the tool used to measure anxiety and fear (Asl et al., 2017; Cianetti et al., 2017). The care provider team needs to intervene with behavior management practices to minimize disruptive behavior that may prevent timely and safe oral health care for these children. Team members who work in a supportive office environment may implement different interventions, often sequentially. Most common are nonpharmacological interventions, including communicative behavior guidance, variants of “tell ‐ show ‐ do,” and distraction techniques (Oliver & Manton, 2015; Prado et al., 2019). Such interventions are effective and carry high societal acceptance but may risk becoming time‐consuming to the detriment of other patients that also require oral care. Theoretically, an uncooperative child can be immobilized by imposing a passive or active restraint, but parental acceptance of such practices has decreased gradually (Eaton et al., 2005; Boka et al., 2014; Patel et al. 2016; Al Zoubi et al., 2021; Ilha et al., 2021), and also reflected in patient rights and safety regulations in many countries (Wells et al., 2018).

The adjunctive use of pharmacological interventions has therefore increased. Individualized clinical circumstances determine whether minimal, moderate, deep sedation or general anesthesia is the optimal choice. However, all forms of sedation comprise latent, albeit small, risks to the patient, and anesthetic gases also present a potential hazard for the clinic staff. Therefore, specialist training and certification are required, along with compulsory equipment on‐site in emergencies (RCDSO, 2018; Ashley et al., 2021). Fundamental questions of resource allocation priorities arise when ultimate objectives such as equal access to oral health care and oral health equity must balance against opportunity costs and sustainable clinical practices within the confines of annual budget allocations to the public oral health care sectors.

Many patients will benefit from receiving operative interventions in general anesthesia given circumstances. However, it has been claimed that undergoing general anesthesia in the dental operatory does not empower apprehensive patients to overcome their anxiety and fear, which predisposes them to future poor oral health and distress (Haworth et al., 2017). Therefore, alternative nonpharmaceutical interventions are needed to create a positive patient effect in dental clinics. One perception is that animal‐assisted interventions can moderate anxiety and fear. Several professional magazines and newsletters have featured practitioners enthusiastically describing how a dog in their clinic helped many patients overcome their anxiety and fear e.g. (Manley, 2016; Prakash, 2016; DeRosier, 2016; Reyes, 2011; Solana, 2015). Two practices have evolved in parallel over the last decade; one is to allow patients to bring along an animal for emotional support or extend such an offer if requested (ADA, 2019). The second approach is dog‐assisted therapy (Cajares et al., 2016).

Although much of the substantial endorsement of animal‐assisted therapy is anecdotal, there is a rationale to explore the claims scientifically. However, multiple difficulties abound when attempting to design methodologically sound clinical studies to study the anxiety‐reducing effects of therapy dogs in healthcare settings (Rodriguez et al., 2021; Santaniello et al., 2020; Waite et al., 2018) and particularly in oral healthcare settings (Schwartz & Patronek, 2002). Beyond satisfying general internal and external validity, clinical trials must describe precise study participant criteria selection and recruitment process, participant age and characteristics of dental anxiety, the rationale for the choice of outcomes and measures, the type of oral health care interventions, and the number of consultations, the dog personality and “competencies” by training, the reasons for videotaping or other forms of recording, consideration of active or passive parents attendance and correcting for the introduction of a therapy animal as a “novelty factor” (Schwartz & Patronek, 2002).

Considering the absence of evidence on dog‐assisted dental therapy in children, we aimed to appraise whether the presence of a certified therapy dog team specially trained for working in an oral health care setting, would facilitate a first‐time clinical examination of referred pediatric patients with anticipatory anxiety and situational fear.

## 2 MATERIALS AND METHODS

### 2.1 Study design

The study was a randomized clinical trial (RCT) with a cross‐over design. The study participants had an intraoral clinical examination twice with a specially trained dental therapy dog present during one of the two examinations. Two specialist pediatric dentists conducted the clinical examination in a separate research operatory room at the Department of Clinical Dentistry, Faculty of Health Sciences, UiT The Arctic University of Norway. The Norwegian Regional Committee for Medical and Health Research Ethics approved a randomized cross‐over trial with a study sample of *n* = 16 children aged between 6 and 12 years (ref. 2017/1078, REC North). According to national patient privacy and health research legislation, all completed case report forms (CRF) were secured at the Department of Clinical Dentistry by the principal investigator. The trial was registered in October 2017 on govclinicaltrials.gov (NCT03324347).

### 2.2 Study participants and recruitment

The Public Dental Service Competence Center of Northern Norway (TkNN) provides specialist services and receives *inter alia* referrals of children with special oral health care needs from the three northernmost provinces of Norway. A pediatric dentist assigned to manage these children informed parents of children with a history of compliance issues due to dental fear, about the possibility of participating in the current trial. Interested parents and their children received letters detailing the information required for study participation consent, guided by The Regional Committee for Medical and Health Research Ethics. The committee advised also that the lower limit for study participation would be set to age 6, considered to be the threshold for understanding the information and correctly sign the forms required for participation consent and completing the questionnaires. The highest age was set to age 12, which is the legal age in Norway when a child participating in a clinical study is required to be accompanied by a parent or a guardian.

The letter addressed to the children consisted primarily of photographs showing the therapy dog in the dental clinic and the anticipated procedures included in the clinical study (Supporting Information: Appendix − Invitation letter). To participate in the trial, the child had previously avoided dental treatment due to dental fear, and both the child and the parent/guardian had to accept the presence of a dental therapy dog in the dental clinic. Exclusion criteria were fearfulness toward dogs by the child or parent/guardian or having a known canine allergy, if the child was immunocompromised, or if the child or parent/guardian could not understand or complete the intended questionnaires. The child and parent were informed in writing and verbally that they could withdraw from the trial at any time without explanation or prejudice.

### 2.3 Data collection and procedures

Nine categories of data were collected, the first three in a waiting room before the clinical examination (child questionnaire, parent questionnaire, and saliva sample), the next three in the operatory room (compliance, skin conductance, and heart rate variability), and the last three in the waiting room following the clinical examination (child‐questionnaire, parent questionnaire, and saliva sample) (Table 1).

**Table 1:** Study progress and data collection (9 categories of data)

	Previsit TKNN/IKO research clinic Recruitment	Visit 1 & Visit 2 IKO research clinic		
		Waiting room**—**before the clinical examination	Operatory room**—**during the clinical examination	Waiting room**—**after the clinical examination
Information and participation consent	x			
Verification of inclusion and exclusion criteria	x			
Reconfirmed participation and further information if needed		x	x	x
Child happy‐sad‐face diagram		1		7
Parent/guardian CFSS‐DS Questionnaire		2		8
Saliva sampling		3		9
Skin conductance			4	
Heart Rate Variability			5	
Patient compliance assessment			6	
A structured observation of therapy dog performance			x	

The child and the parent or guardian were greeted by the principal investigator (A. M. G.) and the examining clinician (K. C. or E. L.) in an empty waiting room, explaining the procedures and answering questions. The parent/guardian completed the CFSS‐DS questionnaire while the child completed the happy‐sad‐face diagram and provided a saliva sample. At the first clinical visit, the study participant selected and opened the opaque envelope containing the study arm allocation.

### 2.4 Randomization and allocation to study arm

The two study arms were to have the dental therapy dog present during the clinical examination on the first versus the second visit. The study arm allocations had been prepared before the trial initiation and concealed in sealed opaque white envelopes. The principal investigator safeguarded the envelopes until the study participant opened the envelope. Opened envelopes were stored as part of the CRF documentation.

If the allocation was to have the dental therapy dog present during the clinical examination on the first visit, the principal investigator went to bring the therapy dog to the waiting room. If the allocation were to have the dental therapy dog present during the second clinical examination, the therapy dog would be present in the waiting room to greet the child and parent at their next visit. In both study arms, the child and therapy dog was given time to become acquainted in the waiting room before proceeding to the operatory room.

### 2.5 Clinical examination

**Figure 1:**
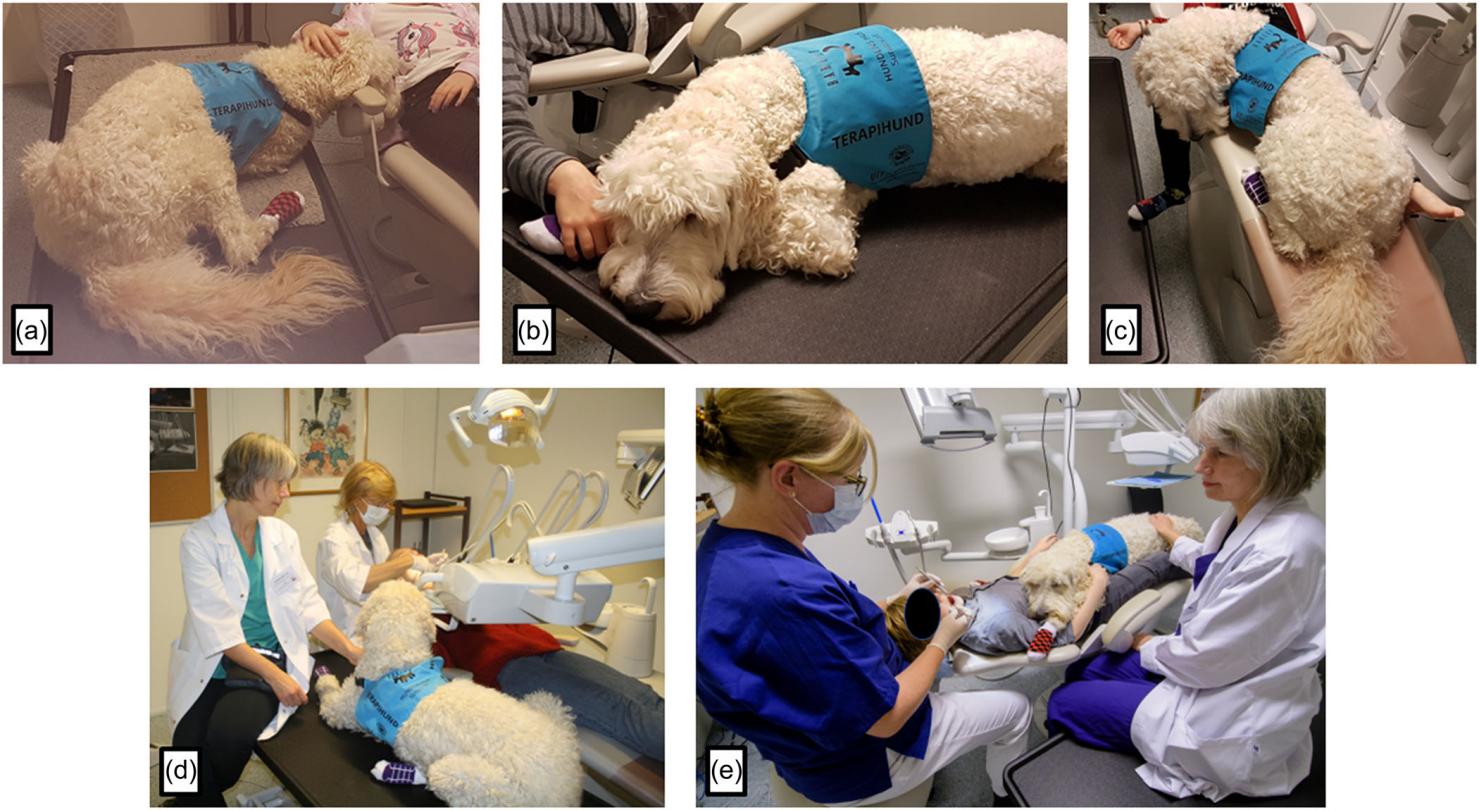
Alternative positions of the dental therapy dog, according to the preference of the study participants. The dog on the veterinary table (a, b, d), alternatively the dog in the dental chair (c, e). (Photos: Anne M. Gussgard [a, b, c, d] Lars Åke Andersen [e])

The child was made comfortable in a conventional dental chair in the operatory room. If a therapy dog was present, the child was empowered to select their preference for interaction. Alternatives were to be on their lap or stay on an electric adjustable veterinary table adjacent to the chair, alternatively resting on the floor (Figure 1).

Next, electrodes for measuring heart rate variability and skin conductance were attached to the child. The electrophysiological equipment was calibrated before the clinician initiated the clinical examination of the child's oral cavity and teeth, using a mirror and probe and a three‐way syringe with water and air. No excavations or other operative interventions were performed, nor were any radiographs made. The parent/guardian was present during the entire clinical examination.

### 2.6 Post examination data collection

Following the clinical examination, the child and parent/guardian returned to the waiting room and completed the post‐examination questionnaires, and the child provided a second saliva sample. The child completed the same happy‐sad‐face diagram as before, while the parent/guardian completed the CFSS‐DS short version questionnaire and was also invited to provide commentaries.

### 2.7 Therapy dog

The dental therapy dog used in this study was a 4‐year‐old 26‐kilo Labradoodle. The dog and handler had undergone a 1‐year therapy dog training program (Hundens hus) and were certified as a therapy dog team for dental clinics. Before each clinic visit, the therapy dog underwent showering, grooming, and claw clipping. Before entering the building, the therapy dog was fitted with a vest describing the dog as such. The dog was fitted with socks with neoprene soles to minimize sliding on slippery surfaces and the risks of accidentally scratching humans. The dog handler (A. M. G.) was consistently present and seated next to the therapy dog for monitoring purposes. The performance and behavior of the dental therapy dog during the encounters with the pediatric patients and their parent/guardian were detailed in writing by the dog handler.

## 3 OUTCOMES

The primary outcome was whether the patient complied during the clinical examination or showed any disruptive behavior (yes/no). The Houpt rating scale for overall behavior (Houpt et al., 1996) is more detailed but was considered unnecessary since a clinical examination is not very invasive and likely not perceived as threatening. “Yes” in the current trial correlates with a score of 6 (excellent, no crying or movement). “No” indicates Houpt scale scores 1−5 (1, terminated, no treatment performed; 2 poor, treatment interrupted and only partially completed; 3 fair, treatment interrupted, but all eventually completed; 4 good, difficult but all treatment performed and 5, very good, some limited crying or movement during sedation onset or mouth prop insertion).

The patient‐ and parent/guarding‐reported outcomes were recorded on different PROM/PREM questionnaires. The child completed a happy‐sad‐face diagram to answer: “How was it like meeting (name of the therapy dog)?”. A score of one was given to a “happy face,” while the outermost “sad face” scored ten.

The parent/guardian was asked to comment on two statements: “I appreciated meeting (name of the therapy dog)” and “I appreciated that the child was supported by (name of the therapy dog)” by completing a 5‐category Likert scale ranging from “completely disagree” to “completely agree.”

Both child and parent/guardian were invited to provide supplemental information by asking the additional question: “Is there anything more you wish to tell us?”.

### 3.1 Psychometric assessments

The child's anticipated anxiety level was assessed using the CFSS‐DS scale (Dental subscale of children's fear survey schedule) completed by the parent or guardian and a happy‐sad‐face diagram completed by the child. The Swedish version of the CFSS‐DS (Klingberg, 1994) was adopted with minor alterations of some words into Norwegian. The questionnaire consisting of 15 items was completed in the waiting room before the clinical examinations. A shorter version consisting of five items was completed following the clinical examination (Supporting Information: Appendix ‐ Table). The items in CFSS‐DS are rated from not afraid (1) to very afraid (5), and the scores are summed without any weighing.

The child was asked two questions, that is, “How do you feel right now?” and “What do you feel about going to the dentist?” by circling on the happy‐sad‐face diagram (Supporting Information: Appendix – Figure 1).

### 3.2 Physiological responses

Saliva samples were collected to measure salivary cortisol levels as potential indicators of anxiety and stress. Saliva was sampled in the waiting room before and after the clinical examination using Salivette® cotton swabs and tubes (Sarstedt). The child chewed 1 min on a cotton swab until the piece became soaked and collected according to the manufacturer's instructions. Collected saliva samples were centrifuged (1000*g*, 2 min) before being divided into two new tubes labeled and stored at −80°C. The samples were analyzed at the Laboratory medicine department at the University Hospital Tromsø (UNN) by use of liquid chromatography coupled with tandem mass spectrometry (Lygre, 2016).

Heart rate variability and skin conductance were measured during the clinical examination using a digital data acquisition system (Biopac MP36R; Biopac Systems, Inc.). Per the manufacturer's instructions, two electrodes were affixed under the foot soles, three to the chest, and connected to wireless recording boxes attached around the ankle and chest. The recorded data were analyzed using dedicated software (AcqKnowledge; version 4.4; Biopac Systems).

## 4 STATISTICS

**Figure 2:**
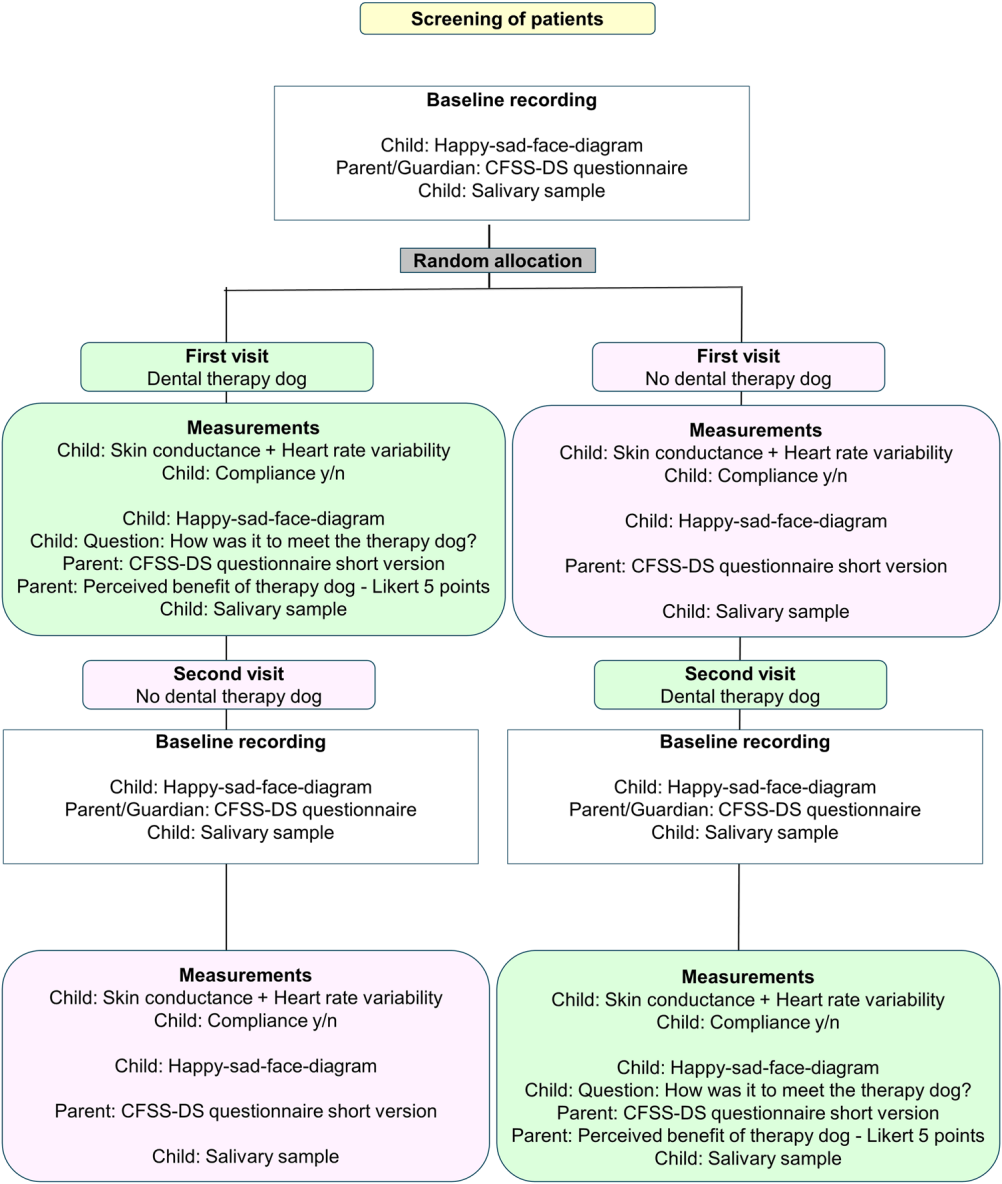
CONSORT flow diagram showing the type of interventions and measurements. (See Table 1 for further details)

The authors failed to identify any relevant published data to calculate study power. The working hypothesis was that high patient compliance would be expected when a dental therapy dog was present in the clinic operatory during the clinical examination and, conversely, less compliance would be expected with no dog presence (Supporting Information: Appendix ‐ Figure 2). A convenience sample size of *n* = 16 study participants was decided in dialog with the local ethics committee. Data analyses were mainly limited to descriptive statistics. Intraindividual percent changes in salivary cortisol levels before and after the clinical examinations were compared with paired *t*‐tests following verification of the normality of the data according to Shapiro−Wilk tests. The data were analyzed using the Statistical Package for the Social Sciences (SPSS) 28.0 (IBM Corp.).

The allocation to interventions and data collection during visit 1 and visit 2 (Table 1) are shown in a CONSORT flow diagram (Figure 2).

## 5 RESULTS

Ten boys and six girls aged between 6 and 12 years (mean age 8.5 years) were recruited, and all completed both clinical examinations conducted between January 2018 and June 2019. The interval between the first and second visits varied from 2 to 13 days (mean 4 days, median 5 days). One examiner (K. C.) saw 10 study participants, and 5 were seen by another (E. L.), while KC saw one participant at the first and EL at the second visit. Most study participants (*n* = 11) choose to have the therapy dog on the veterinary table adjacent to the patient's chair with or without the dog resting its head or snout on the armrest or the patient's arm. Four preferred to have the dog on their lap part of the time and after that on the veterinary table. In contrast, one participant had the therapy dog on the lap throughout the entire clinical examination process.

**Figure 3:**
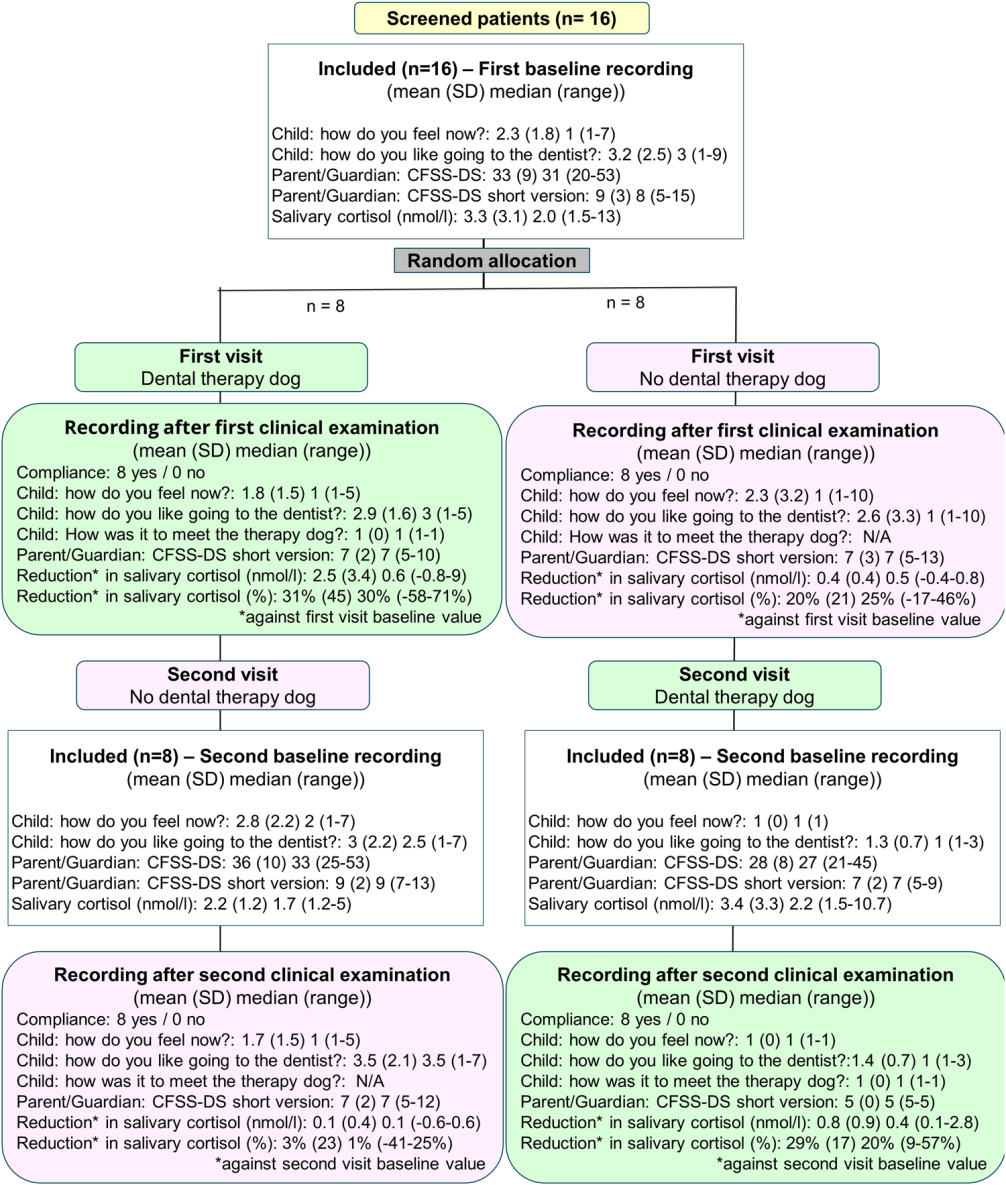
CONSORT flow diagram showing the data for the primary and secondary outcomes

All the study participants complied satisfactorily without any disruptive behaviors while undergoing the clinical examinations, regardless of whether or not the dental therapy dog was present in the operatory room (Figure 3).

All study participants reported maximum scores for satisfaction after having undergone the clinical examination with the presence of the dental therapy dog in the clinic operatory. Moreover, all parents/guardians with one exception who scored “2” scored “5,” that is, agree, upon answering the statement: “I appreciated that the child was supported by (name of the therapy dog),” and 13 of 16 parents/guardians scored “5,” that is, agree, upon answering the: “I appreciated meeting (name of the therapy dog)” whereas 3 of 16 parents/guardians scored “4” on the same statement.

### 5.1 Psychometric assessments

Before the first clinical examination, the parent‐reported CFSS‐DS scores ranged between 20 and 53 (mean 33, standard deviation [SD] 9). New baseline scores before the clinical examination at the second visit differed slightly for the cohort that had undergone clinical examination in the presence of the therapy dog (mean 36 [SD 10]) versus the second cohort of children about to meet the therapy dog for the first time (mean 28 [SD 8]) (Figure 3).

The divergence also appears in the CFSS‐DS short version scores. The cohort that had the therapy dog present during the clinical examination on the first visit reported scores between 5 and 12 after the clinical examination on the second visit (mean 7 [SD 2]). In contrast, the scores in the cohort that did not meet the therapy dog at the clinical examination on their first visit were all “5,” that is, not afraid at all.

Interestingly, the same tendency is also reflected in the child‐reported happy‐sad‐face scores. that is, the mean scores of 2.3 and 3.2 at baseline before the first clinical examination jumped to 2.8 and 3.0 before the examination on the second visit in the cohort that had undergone the clinical examination on the first visit in the therapy dog's presence. In contrast, the values dropped to 1.0 and 1.3 in the cohort of children about to meet the therapy dog for the first time (Figure 3).

### 5.2 Physiological responses

The salivary cortisol level ranged between 1.5 and 13 nmol/l (mean 3.3 [SD 3.1]) at the baseline recordings at the first visit. During the clinical examination on the second visit, the mean cortisol level decreased by 2.5 nmol/l (31% [SD 45] range −58 to 71%) in the cohort with the dog present at the first visit and 0.4 nmol/l (20% [SD 21] range −17 to 46%) without the therapy dog presence. In the baseline recording at the second visit, the salivary cortisol level ranged between 1.2 and 10.7 nmol/l, with minor differences between the two cohorts. The cohort examined in the presence of a therapy dog had a mean of 2.2 nmol/l (SD 1.2) compared to 3.4 nmol/l (SD 3.3) in the cohort about to meet the dental therapy dog at their second visit. At the second clinical examination, the mean cortisol level decreased by 0.8 nmol/l (29% [SD 17] range 9 to 57%) in the cohort with the dog present and 0.1 nmol/l (3% [SD 23] range −41 to 25%) without the therapy dog presence. Intraindividual variation was noted. Two study participants showed higher cortisol levels than the others, attributed to the effects of early puberty, that is, both were girls aged 12.

The Shapiro−Wilk tests did not show significant deviation from the normality of the data within the four comparisons of percentage salivary cortisol changes, that is, *W*(8) = 0.842 (*p* = .089); 0.904 (*p* = .345); 0.954, (*p* = .835) and 0.864 (*p* = .141).

The salivary cortisol levels decreased significantly during the first clinical examination both in the presence of the therapy dog (*t* = 4.7, *p* = .006) and without the presence of the therapy dog (*t* = 2.5, *p* = .04). The same effect also occurred during the second clinical examination when the therapy dog was present in the clinic (*t* = 4.9, *p* = .002).

### 5.3 Electrophysiological measurements

The recorded heart rate variability and skin conductance data were analyzed with the dedicated software. However, the data contained much noise and artefacts to determine heart rate variability reliably. The skin conductance electrode signals were also very weak, which made the analyses challenging. Due to the questionable measurement reliability and uncertain validity, it was determined not to attempt to interpret the electrophysiological measurement data.

## 6 DISCUSSION

### 6.1 Study methodology

A cross‐over study design was selected to avoid a more extensive than necessary study sample of a vulnerable patient group. Different data would likely have emerged if the study had been planned as a parallel RCT, where the children in one study arm would have been denied the possibility of meeting the therapy dog. A parallel RCT design would likely have required a large sample size based on a power calculation to account for potential confounding variables, including but not limited to variance in cognitive abilities, temperament, earlier experience of dental treatment, and extent of parental support. There is little guidance from systematic reviews on whether parallel or cross‐over RCTs are preferred for studying the effects of animal‐assisted therapy (Feng et al., 2021; Rodriguez et al., 2021; Santaniello et al., 2020; Waite et al., 2018; Zhang et al., 2021).

A potential shortcoming of this trial in terms of the possible risk of confounding is a relatively short “washout period” between the two clinical examinations. The mean interval between the first and second visit was 2 days, which was intentionally selected not to be too long. The rationale was that we wanted to enable the children to start their treatment in the specialist clinic as soon as possible, considering a possible delay in the remittance process.

### 6.2 Study participants and study invitation

The targeted study population was “pediatric patients with anticipatory anxiety and situational fear.” The baseline CFSS score of the study participants at the trial start ranged from 20 to 53 (mean 33 [SD 9]), which may be considered moderate fear. However, different cut‐off thresholds have been used to differentiate between high or low dental fear. A score of 38 is regarded as high fear, while other thresholds have been assigned to low and moderate fear or anxiety. A Norwegian study relevant to the current trial used a CFSS‐DS score of 29 as a threshold for high anxiety (Raadal et al., 2002).

A weakness of this trial in terms of the generalizability of the findings is that the study participants were self‐selecting, typical for clinical studies involving assistance animals. Using animal‐assisted therapy is unrealistic for patients who are indifferent to animals and afraid at worst. In the current trial, the study participants knew that they, in any case, would be able to meet the dental therapy dog if not during the first clinical consultation, then in the second.

### 6.3 Therapy dogs in clinic operatories

Perhaps contrary to many beliefs, former dogmatic opinions on this question (Schienbaum, 2011) are today primarily resolved. Over the last few decades, many countries have introduced legislation that allows disabled individuals the legal right to bring their assistance dogs into dental clinics (ADA, 1992; Poplett, 2018). In parallel, the global trend of patients requesting to be accompanied by their private dog for emotional support prompted concerns about both patients and clinicians misunderstanding different assistance animals' legal rights (AVMA, 2017). Several guidance articles describe the best legal, ethical, and professional practices regarding different categories of assistance dogs in oral healthcare clinics (ADA, 2019; AVMA, 2019; CDA, 2018; Schulte, 2017).

Among the alternatives of having an emotional support dog versus a trained therapy dog, one needs to realize that the care provided in an oral health clinic is a unique treatment situation. A patient rest inclined in a confined space with little maneuverability surrounded by atypical smell, chemicals, aerosols, noise, and occasional high work activity, that is, hazards associated with occupational health‐related problems known to dental clinic staff members (Moodley et al., 2018). These and further hazards also apply to any dog present in the clinic (Gussgard et al., 2019b). Unless the dog has undergone proper priming to function under taxing conditions in a dental clinic, the dog risks becoming stressed and disruptive, an additional hazard to those present in the clinic (Gussgard et al., 2019a). A dog that has been scared under such conditions will attempt to avoid all future resemblances of similar encounters. Dog owners should seriously consider the need to bring along their pets from an animal welfare perspective.

### 6.4 Primary outcome

This trial was planned for an anticipated excellent study participant compliance in the presence of a therapy dog during the first clinical examination, in contrast to more negative compliance with no dog present (Supporting Information: Appendix ‐ Figure 2). However, contrary to expected, all study participants complied fully during both clinical examinations, with or without the dog. Possible explanations remain speculative, starting with why some children develop anticipatory anxiety and situational fear formed by a complex interplay of several cognitive and noncognitive factors (Scandurra et al., 2021) and influenced by parental behavior (Chapman & Kirby‐Turner, 2018).

Apart from a latent concern that the parent/guardian‐reported CFSS‐DS scores were possibly inflated, it cannot be ruled out that the study invitation letter may have impacted the findings. The detailed descriptions and pictures of a dental examination and a dental therapy dog likely triggered emotional reactions of interest and curiosity. They created new and favorable anticipations and associations before the first dental examinations. In turn, the first positive encounter with a new dentist, with or without the presence of a dog, created new experiences, where the study participants and or their guardians/parents approached the second dental visit with interest/curiosity rather than anxiety/fear that the dental appointments had elicited in their past.

This hypothesis is supported by the CFSS‐DS data (Figure 3), which show that the children who overcame first a clinical examination without the presence of a therapy dog were encouraged by this experience and showed even fewer signs and symptoms of anticipatory anxiety and situational fear before and after their second clinical examination in the presence of a therapy dog.

### 6.5 Secondary outcomes

In contrast to the patient‐ and parent/guardian reported outcomes and experiences in this trial indicating a positive effect of having a therapy dog present, the selected psychometric measurements were more variable. The biological variance likely impacted this small sample's physiological responses. Similar observations have also been made in recent publications reporting on the experiences with the use of therapy dogs in the oral health care of pediatric patients (Vincent et al., 2020).

A challenge in a clinical study on this theme is identifying the most appropriate objective and subjective measures of anxiety and fear reduction among pediatric patients. There are no gold standards amongst the many reported physiologic (also known as “objective”) outcomes, including heart rate, blood pressure, skin temperature, electrodermal activity, pulse oximetry, and different saliva biomarkers such as cortisol, alpha‐amylase, and oxytocin (Guinot Jimeno et al., 2011).

Psychometric anxiety assessment has been predominantly assessed using STAI‐CH (State‐Trait anxiety inventory for children) in different healthcare settings (Feng et al., 2021; Zhang et al., 2021). However, these have rarely been used in oral health care where instead the CFSS‐DS scale (Children's Fear Survey Schedule‐Dental Subscale) (Cuthbert & Melamed, 1982) for some years has been the most favored tool in dentistry (Asl et al., 2017; Cianetti et al., 2017; Klingberg & Broberg, 2007).

The heart rate variability and skin conductance were measured according to the manufacturer's instructions. However, the recorded data could not be analyzed reliably. One reason was that the electrodes ideally should have been placed in good time before the clinical examination to allow ample time for the adaptation between the skin, the conductance gel, and the electrodes. In this study, the children were not seated in the dental chair longer than necessary to undertake the clinical examination. Moreover, frequent micro and macro movements while sitting in the dental chair caused signaling noise and artifacts. In retrospect, the selected electrophysiological measurements seem not ideal for measuring child anxiety in a dental operatory setting.

### 6.6 Findings

The general finding of this pilot study is that all study participants succeeded in collaborating in the clinical examinations, despite their history of noncompliance. This finding applies only to the specific subgroup of individuals, that is, the study population of referred pediatric patients with anticipatory anxiety and situational fear that undergo clinical examination by a new dentist. It is uncertain how the findings can be extrapolated to other individuals with anticipatory anxiety and situational fear undergoing different interventions. Adults with dental anxiety and fear may include both individuals with previous unpleasant or painful experiences in the dental chair, with self‐reported poor oral health, or with injection phobia or avoidance behaviors, and even with different psychiatric disorders, including trait anxiety and phobic anxiety (Strøm et al., 2020).

A potential criticism of the current trial is that the study participants did not receive any operative interventions beyond a clinical examination. The explanation is that there were no reports of dog‐assisted trials in pediatric dental settings in 2016 when the current trial was planned. It seemed logical that a first step would be to assess the study participant's behavior while conducting a repeatable intervention, that is, a routine clinical intraoral examination. It is important that referred pediatric patients with anticipatory anxiety and situational fear undergo a first‐time clinical examination by a clinician in a timely and calm manner. Such conditions likely enable more referred patients to be examined within set time frames and resource settings.

Future trials should address the possible benefits of dog assistance not only during clinical examinations but also in particular treatment situations. There is still only very limited data on this study domain (Charowski et al., 2021; Cruz‐Fierro et al., 2019; Nammalwar & Rangeeth, 2018; Thakkar et al., 2021; Vincent et al., 2020). Nevertheless, the number of clinical studies on this theme is limited. The few studies that have been published are small and vary considerably in patient characteristics, reported outcomes, and choice of a therapy dog. One also needs to consider the possibility of publication bias in the research literature.

## 7 ADDENDUM

The clinician (E. L.) that recruited the study participants in the dental public health clinic has provided intriguing feedback that the children who partook in the trial demonstrated remarkable positive changes in how they subsequently coped with their dental appointments. Investigators should consider implementing such outcomes in future clinical study protocols, given that PROMS and PREMS have become important considerations when assessing qualities of care provision.

## 8 CONCLUSION

The presence of a dental therapy dog in an operatory room facilitates intraoral clinical examinations of pediatric patients referred to a specialist clinic due to anticipatory anxiety and situational fear.

The use of a dental therapy dog in such a context was endorsed by PROMs and PREMs from the study participants and their parents/guardians. At the same time, the psychometric measures and physiological responses were indeterminate.

An offer to have a trained dental therapy dog accessible in the dental operatory for new patients with anticipatory anxiety and situational fear may shorten the time required to conduct a clinical examination, enabling more referred patients to be examined within set time frames and limited resource settings.

## AUTHOR CONTRIBUTIONS

Anne M. Gussgard and Kerstin Carlstedt: Participant recruitment, clinical examination, data collection and measurements. Anne M. Gussgard: Ethics approval. Anne M. Gussgard, Kerstin Carlstedt, and Malin Meirik: Research design, manuscript preparation and final manuscript review.

## CONFLICT OF INTEREST

The authors declare no conflict of interest.

## Supporting information

Supporting information.

## Data Availability

The data that support the findings of this study are available from the corresponding author upon reasonable request.

## References

[cre2679-bib-0001] ADA, American Dental Association . (1992). Americans with Disabilities Act: questions & answers. Journal of the American Dental Association, 123(Suppl), 1–12. 10.14219/jada.archive.1992.0255 1530821

[cre2679-bib-0002] ADA, American Dental Association Council on Ethics, Bylaws and Judicial Affairs . (2019). The ethics of emotional support animals in the dental office. The Journal of the American Dental Association, 150, 982–984. https://jada.ada.org/article/S0002-8177(19)30509-4/fulltext 31668174 10.1016/j.adaj.2019.07.020

[cre2679-bib-0051] Al Zoubi, L. , Schmoeckel, J. , Mustafa Ali, M. , & Splieth, C. H. (2021). Parental acceptance of advanced behaviour management techniques in paediatric dentistry in families with different cultural background. European Archives of Paediatric Dentistry, 22, 707–713. 10.1007/s40368-021-00607-4 33768499 PMC8302555

[cre2679-bib-0052] Ashley, P. , Anand, P. , & Andersson, K. (2021). Best clinical practice guidance for conscious sedation of children undergoing dental treatment: An EAPD policy document. European Archives of Paediatric Dentistry, 28, 989–1002. 10.1007/s40368-021-00660-z PMC8629790 34453697

[cre2679-bib-0003] Asl, A. N. , Shokravi, M. , Jamali, Z. , & Shirazi, S. (2017). Barriers and drawbacks of the assessment of dental fear, dental anxiety and dental phobia in children: A critical literature review. Journal of Clinical Pediatric Dentistry, 41, 399–423. 10.17796/1053-4628-41.6.1 28937891

[cre2679-bib-0004] AVMA, American Veterinary Medical Association . (2017). *Assistance animals: Rights of access and the problem of fraud*. Retrieved April 21, 2017, From https://www.avma.org/KB/Resources/Reports/Documents/Assistance-Animals-Rights-Access-Fraud-AVMA.pdf

[cre2679-bib-0005] AVMA, American Veterinary Medical Association . (2019). Service, emotional support and therapy animals. https://www.avma.org/resources-tools/animal-health-welfare/service-emotional-support-and-therapy-animals

[cre2679-bib-0053] Boka, V. , Arapostathis, K. , Vretos, N. , & Kotsanos, N. (2014). Parental acceptance of behaviour‐management techniques used in paediatric dentistry and its relation to parental dental anxiety and experience. European Archives of Paediatric Dentistry, 15, 333–339. 24676547 10.1007/s40368-014-0119-y

[cre2679-bib-0006] Cajares, C. , Rutledge, C. , & Haney, T. (2016). Animal assisted therapy in a special needs dental practice: An interprofessional model for anxiety reduction. Journal of Intellectual Disability—Diagnosis and Treatment, 4, 25–28. 10.6000/2292-2598.2016.04.01.3

[cre2679-bib-0008] CDA, California Dental Association . (2018). Service animal, comfort animal or pet? What to know about bringing animals into the dental practice. CDA update, 30(11), 10. https://www.cda.org/Portals/0/update/update_112018.pdf

[cre2679-bib-0009] Chapman, H. R. , & Kirby‐Turner, N. (2018). Psychological intrusion—an overlooked aspect of dental fear. Frontiers in Psychology, 9, 501. 10.3389/fpsyg.2018.00501 29719519 PMC5913370

[cre2679-bib-0010] Charowski, M. , Wells, M. H. , Dormois, L. , Fernandez, J. A. , Scarbecz, M. , & Maclin, M. (2021). A randomized controlled pilot study examining effects of animal assisted therapy in children undergoing sealant placement. Pediatric Dentistry, 43, 10–16. 33662243

[cre2679-bib-0011] Cianetti, S. , Lombardo, G. , Lupatelli, E. , Pagano, S. , Abraha, I. , Montedori, A. , Caruso, S. , Gatto, R. , De Giorgio, S. , & Salvato, R. (2017). Dental fear/anxiety among children and adolescents. A systematic review. European journal of paediatric dentistry, 18(2), 121–130. 10.23804/ejpd.2017.18.02.07 28598183

[cre2679-bib-0012] Cruz‐Fierro, N. , Vanegas‐Farfano, M. , & González‐Ramírez, M. T. (2019). Dog‐Assisted therapy and dental anxiety: A pilot study. Animals: An open access journal from MDPI, 9, 512. 10.3390/ani9080512 31370328 PMC6720307

[cre2679-bib-0013] Cuthbert, M. I. , & Melamed, B. G. (1982). A screening device: Children at risk for dental fears and management problems. ASDC Journal of Dentistry for Children, 49, 432–436. 6960031

[cre2679-bib-0054] DeRosier, J. (2016). Dental therapy dogs. Canman's best friend help your practice? CDS Review, 109, 10–12. 29694741

[cre2679-bib-0055] Eaton, J. J. , McTigue, D. J. , & Fields, H. W. Jr. , & Beck, M. (2005). Attitudes of contemporary parents toward behavior management techniques used in pediatric dentistry. Pediatric Dentistry, 27, 107–113. 15926287

[cre2679-bib-0014] Feng, Y. , Lin, Y. , Zhang, N. , Jiang, X. , & Zhang, L. (2021). Effects of animal‐assisted therapy on hospitalized children and teenagers: A systematic review and meta‐analysis. Journal of Pediatric Nursing, 60, 11–23. 10.1016/j.pedn.2021.01.020 33582447

[cre2679-bib-0015] Guinot Jimeno, F. , Yuste Bielsa, S. , Cuadros Fernández, C. , Lorente Rodríguez, A. I. , & Mercadé Bellido, M. (2011). Objective and subjective measures for assessing anxiety in paediatric dental patients. European journal of paediatric dentistry, 12(4), 239–244. https://admin.ejpd.eu/download/2011-04-07.pdf 22185248

[cre2679-bib-0016] Gussgard, A. M. , Weese, J. S. , Hensten, A. , & Jokstad, A. (2019a). Dog‐assisted therapy in the dental clinic: Part A‐Hazards and assessment of potential risks to the health and safety of humans. Clinical and Experimental Dental Research, 5, 692–700. 10.1002/cre2.240 31890307 PMC6934338

[cre2679-bib-0017] Gussgard, A. M. , Weese, J. S. , Hensten, A. , & Jokstad, A. (2019b). Dog‐assisted therapy in the dental clinic. Part B. Hazards and assessment of potential risks to the health and safety of the dental therapy dog. Clinical and Experimental Dental Research, 5, 701–711. 10.1002/cre2.239 31890308 PMC6934346

[cre2679-bib-0018] Haworth, S. , Dudding, T. , Waylen, A. , Thomas, S. J. , & Timpson, N. J. (2017). Ten years on: Is dental general anaesthesia in childhood a risk factor for caries and anxiety? British Dental Journal, 222, 299–304. 10.1038/sj.bdj.2017.175 28232699 PMC5565940

[cre2679-bib-0019] Houpt, M. I. , Kupietzky, A. , Tofsky, N. S. , & Koenigsberg, S. R. (1996). Effects of nitrous oxide on diazepam sedation of young children. Pediatric Dentistry, 18, 236–241. 8784916

[cre2679-bib-0057] Ilha, M. C. , Feldens, C. A. , Razera, J. , Vivian, A. G. , de Rosa Barros Coelho, E. M. , & Kramer, P. F. (2021). Protectivestabilization in pediatric dentistry: A qualitative study on the perceptions ofmothers, psychologists, and pediatric dentists. International Journal of Paediatric Dentistry, 31, 647–656. 33220112 10.1111/ipd.12751

[cre2679-bib-0020] Klingberg, G. (1994). Reliability and validity of the Swedish version of the dental subscale of the children's fear survey schedule, CFSS‐DS. Acta Odontologica Scandinavica, 52, 255–256. 10.3109/00016359409029055 7985512

[cre2679-bib-0021] Klingberg, G. , & Broberg, A. G. (2007). Dental fear/anxiety and dental behaviour management problems in children and adolescents: A review of prevalence and concomitant psychological factors. International Journal of Paediatric Dentistry, 17(6), 391–406. 10.1111/j.1365-263X.2007.00872.x 17935593

[cre2679-bib-0022] Lygre, H. (2016). Quantification of cortisol in saliva. The Norwegian Dental Journal, 126, 684–687.

[cre2679-bib-0023] Manley, L. (2016). On the use of pets to manage dental anxiety. Dental Hypotheses, 7(3), 117. 10.4103/2155-8213.190518

[cre2679-bib-0024] Moodley, R. , Naidoo, S. , & Wyk, J. (2018). The prevalence of occupational health‐related problems in dentistry: A review of the literature. Journal of Occupational Health, 60, 111–125. 10.1539/joh.17-0188-RA 29213011 PMC5886878

[cre2679-bib-0025] Nammalwar, R. , & Rangeeth, P. (2018). A bite out of anxiety: Evaluation of animal‐assisted activity on anxiety in children attending a pediatric dental outpatient unit. Journal of Indian Society of Pedodontics and Preventive Dentistry, 36, 181–184. 10.4103/JISPPD.JISPPD_54_18 29970636

[cre2679-bib-0026] Oliver, K. , & Manton, D. J. (2015). Contemporary behavior management techniques in clinical pediatric dentistry: Out with the old and in with the new? Journal of Dentistry for Children (Chicago, Ill.), 82, 22–28. 25909839

[cre2679-bib-0058] Patel, M. , McTigue, D. J. , Thikkurissy, S. , & Fields, H. W. (2016). Parental attitudes toward advanced behavior guidance techniques used in pediatric dentistry. Pediatric Dentistry, 38, 30–36. 26892212

[cre2679-bib-0059] Poplett, E. (2018). Puppy professor getspaws‐itive reviews. UNC, Chapel Hill.

[cre2679-bib-0027] Prado, I. M. , Carcavalli, L. , Abreu, L. G. , Serra‐Negra, J. M. , Paiva, S. M. , & Martins, C. C. (2019). Use of distraction techniques for the management of anxiety and fear in paediatric dental practice: A systematic review of randomized controlled trials. International Journal of Paediatric Dentistry, 29, 650–668. 10.1111/ipd.12499 30908775

[cre2679-bib-0028] Prakash, A. (2016). Meet the dog who makes kids actually LIKE going to the dentist. *TODAY Newsletter*. https://www.today.com/pets/meet-dog-who-makes-kids-actually-going-dentist-t84496

[cre2679-bib-0029] Raadal, M. , Strand, G. V. , Amarante, E. C. , & Kvale, G. (2002). Relationship between caries prevalence at 5 years of age and dental anxiety at 10. European journal of paediatric dentistry, 3(1), 22–26. 12871013

[cre2679-bib-0060] RCDSO, Royal College of Dental Surgeons of Ontario . (2018). Standard of practice on the use of sedation and general anesthesia in dental practice. Royal College of Dental Surgeons of Ontario.

[cre2679-bib-0030] Reyes, P. (2011). Pooch in the practice: Mona Lisa makes patients smile. Journal of the California Dental Association, 39, 137–139. 21563593

[cre2679-bib-0031] Rodriguez, K. E. , Herzog, H. , & Gee, N. R. (2021). Variability in Human‐Animal interaction research. Frontiers in Veterinary Science, 7, 619600. 10.3389/fvets.2020.619600 33521092 PMC7843787

[cre2679-bib-0032] Santaniello, A. , Dicé, F. , Claudia Carratú, R. , Amato, A. , Fioretti, A. , & Menna, L. F. (2020). Methodological and terminological issues in animal‐assisted interventions: An umbrella review of systematic reviews. Animals: An Open Access Journal from MDPI, 10(5), 759. 10.3390/ani10050759 32349351 PMC7277107

[cre2679-bib-0033] Scandurra, C. , Gasparro, R. , Dolce, P. , Bochicchio, V. , Muzii, B. , Sammartino, G. , Marenzi, G. , & Maldonato, N. M. (2021). The role of cognitive and non‐cognitive factors in dental anxiety: A mediation model. European Journal of Oral Sciences, 129(4), e12793. 10.1111/eos.12793 33945646 PMC8453836

[cre2679-bib-0034] Schienbaum, R. P. (2011). Spotlight, dog in the practice are ‘unacceptable’. Journal of the California Dental Association, 39, 289. https://www.cda.org/Portals/0/journal/journal_052011.pdf 21721472

[cre2679-bib-0035] Schulte, D. (2017). When should I allow patients to bring dogs into my office? The Journal of the Michigan Dental Association, 99, 18. https://www.michigandental.org/legal-article/when-should-i-allow-patients-to-bring-dogs-into-my-office/ 30398310

[cre2679-bib-0036] Schwartz, A. , & Patronek, G. (2002). Methodological issues in studying the anxiety‐reducing effects of animals: Reflections from a pediatric dental study. Anthrozoös, 15, 290–299. 10.2752/089279302786992432

[cre2679-bib-0037] Solana, K. (2015). Pediatric dentists shares dental therapy dog success story. *ADA News*. https://www.ada.org/en/publications/ada-news/2015-archive/may/pediatric-dentist-shares-dental-therapy-dog-success-story

[cre2679-bib-0038] Strøm, K. , Skaare, A. B. , & Willumsen, T. (2020). Dental anxiety in 18‐year‐old Norwegians in 1996 and 2016. Acta Odontologica Scandinavica, 78(1), 13–19. 10.1080/00016357.2019.1637933 31287346

[cre2679-bib-0039] Thakkar, T. K. , Naik, S. N. , & Dixit, U. B. (2021). Assessment of dental anxiety in children between 5 and 10 years of age in the presence of a therapy dog: A randomized controlled clinical study. European Archives of Paediatric Dentistry, 22, 459–467. 10.1007/s40368-020-00583-1 33245525

[cre2679-bib-0040] Vincent, A. , Heima, M. , & Farkas, K. J. (2020). Therapy dog support in pediatric dentistry: A social welfare intervention for reducing anticipatory anxiety and situational fear in children. Child and Adolescent Social Work Journal, 37, 615–629. 10.1007/s10560-020-00701-4

[cre2679-bib-0041] Waite, T. C. , Hamilton, L. , & O'Brien, W. (2018). A meta‐analysis of animal assisted interventions targeting pain, anxiety, and distress in medical settings. Complementary therapies in clinical practice, 33, 49–55. 10.1016/j.ctcp.2018.07.006 30396626

[cre2679-bib-0061] Wells, M. H. , Dormois, L. D. , & Townsend, J. A. (2018). Behavior guidance: That was then but this is now. General Dentistry, 66, 39–45. 30444705

[cre2679-bib-0042] Zhang, Y. , Yan, F. , Li, S. , Wang, Y. , & Ma, Y. (2021). Effectiveness of animal‐assisted therapy on pain in children: A systematic review and meta‐analysis. International journal of nursing sciences, 8(1), 30–37. 10.1016/j.ijnss.2020.12.009 33575442 PMC7859554

