# Supplementary material for: Intraoral clinical examinations of pediatric patients with anticipatory anxiety and situational fear facilitated by therapy dog assistance: A pilot RCT
Source: Clin Exp Dent Res. 2022 Oct 19;9(1):122–33. doi: 10.1002/cre2.679 (PMC9932233; doi:10.1002/cre2.679)
Supplement: Supplementary file 1 — Supporting information. [file CRE2-9-122-s001.pdf]

Appendix - Invitation letter, page 1 of 2

Terapihund til hjelp ved tannbehandling

Hunden Barley kan bli med barn til tannlegen

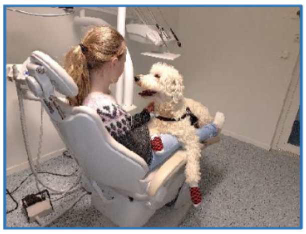
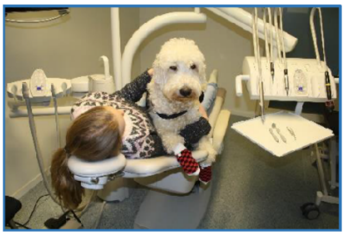
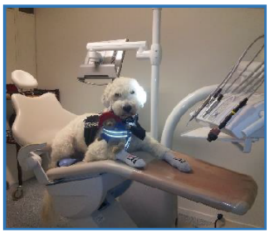

HVORFOR BLIR DU SPURT OM Å VÆRE MED?

Vi vil undersøke om barn synes det er bedre å gå til tannlegen hvis det er en hund tilstede i tannlegekontoret.

HVA VIL SKJE DERSOM DU DELTAR?

Du vil besøke oss to ganger. Den ene gangen vil du møte hunden Barley. Barley vil da bli med deg på besøket i tannlegekontoret.

Tannlegene Kerstin Carlstedt og Anne M. Gussgard vil hilse på deg på venteværelset i 4. etasje i Tannbygget.

På venteværelset:

1. Vi vil be deg om å sette kryss på et spørreskjema.

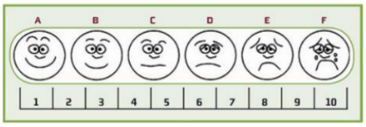

2. Vi vil be om en spyttprøve. Det betyr at du tygger på en liten bommulsbit i ett minutt, og så puttes bomullsbiten ned i et lite rør.

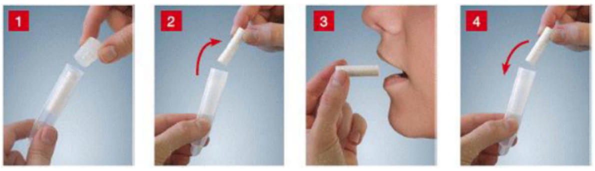

3. En av de gangene du besøker oss, vil hunden Barley hilse på deg på venteværelset, og så vil han bli med deg inn på tannlegekontoret.

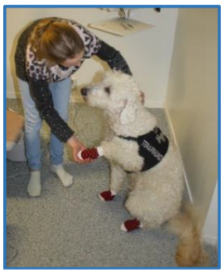

Appendix - Invitation letter, page 2 of 2

Terapihund til hjelp ved tannbehandling

Inne på tannlegekontoret:

4. Du vil få noen små limputer under foten din og på brystet ditt og
   - rundt ankelen og rundt armen får du en liten ledningsboks, slik som du ser på bildene

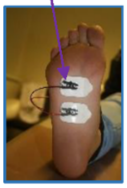
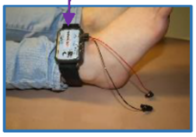

5. Tannlege Kerstin Carlstedt vil undersøke tennene dine

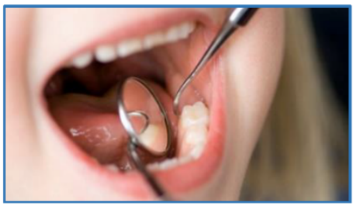
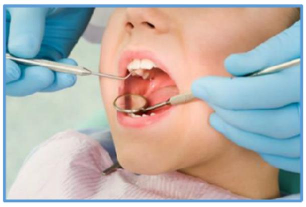

6. Hvis hunden Barley er med på besøket i tannlegekontoret, så kan du bestemme om Barley skal sitte eller ligge ved siden av deg i tannlegestolen eller på sitt eget bord. Han kan også bare være på gulvet ved siden av tannlegestolen.

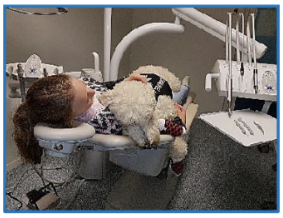
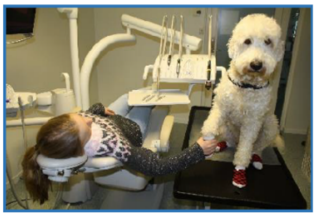
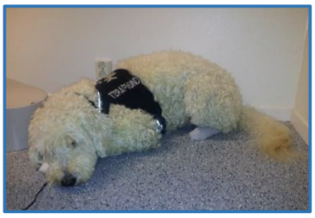

Etter besøket på tannlegekontoret:

Når du er ferdig på tannlegekontoret, går vi tilbake til venteværelset, og da ber vi deg om at du fyller ut spørreskjemaet en gang til. Vi ber deg også om enda en spyttprøve.

Besøket hos oss varer ca. 30 minutter og du kan ta med deg mamma, pappa eller en annen voksen person når du kommer til oss.

HVA VIL SKJE DERSOM DU IKKE DELTAR

Det er frivillig å delta i denne undersøkelsen. Du bestemmer selv om du har lyst til å være med og møte hunden Barley.

Appendix – Table

Appendix -Table. Parent/Guardian assessment of child anxiety and use of a therapy dog. A. *CFSS-DS before the clinical examination (max score = 75), B. Subset of CFSS-DS after the clinical examination (max score = 25).*

A

How afraid is your child of..................?

		**Not afraid at all**	**A little afraid**	**A fair amount afraid**	**Pretty much afraid**	**Very afraid**
1	Dentists					
2	Doctors					
3	Injection (shots)					
4	Having somebody examine your mouth					
5	Having to open your mouth					
6	Having a stranger touch you					
7	Having somebody look at you					
8	The dentist drilling					
9	The sight of the dentist drilling					
10	The noise of the dentist drilling					
11	Having somebody put instruments in your mouth					
12	Choking					
13	Having to go to the hospital					
14	People in white uniforms					
15	Having the nurse clean your teeth					

B

How afraid of ……………do you think your son/daughter has been today?

		**Not afraid at all**	**A little afraid**	**A fair amount afraid**	**Pretty much afraid**	**Very afraid**
**1**	Dentist					
**2**	Having somebody examine your mouth					
**3**	Having to open your mouth					
**4**	Having a stranger touch you					
**5**	Having somebody look at you					

Appendix – Figure 1

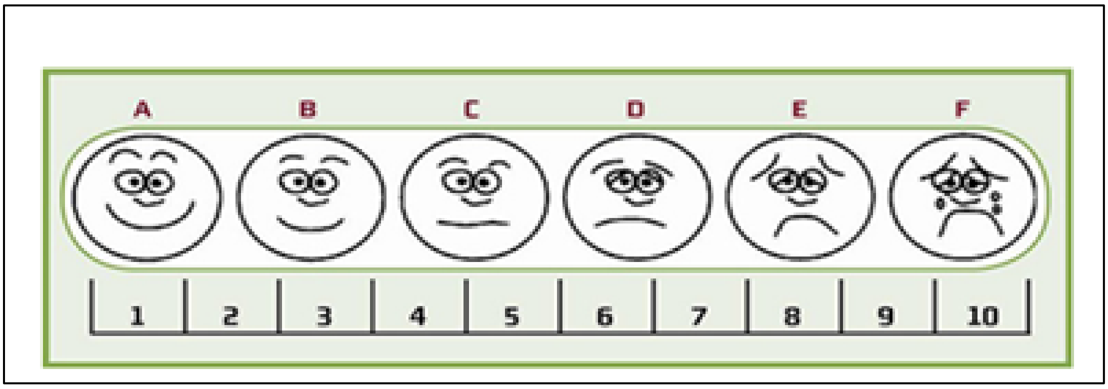

Figure legend:

The child completed a happy-sad face diagram to illustrate their emotion after interacting with the therapy dog and describe their anxiety level before and after the clinical examination.

Appendix – Figure 2

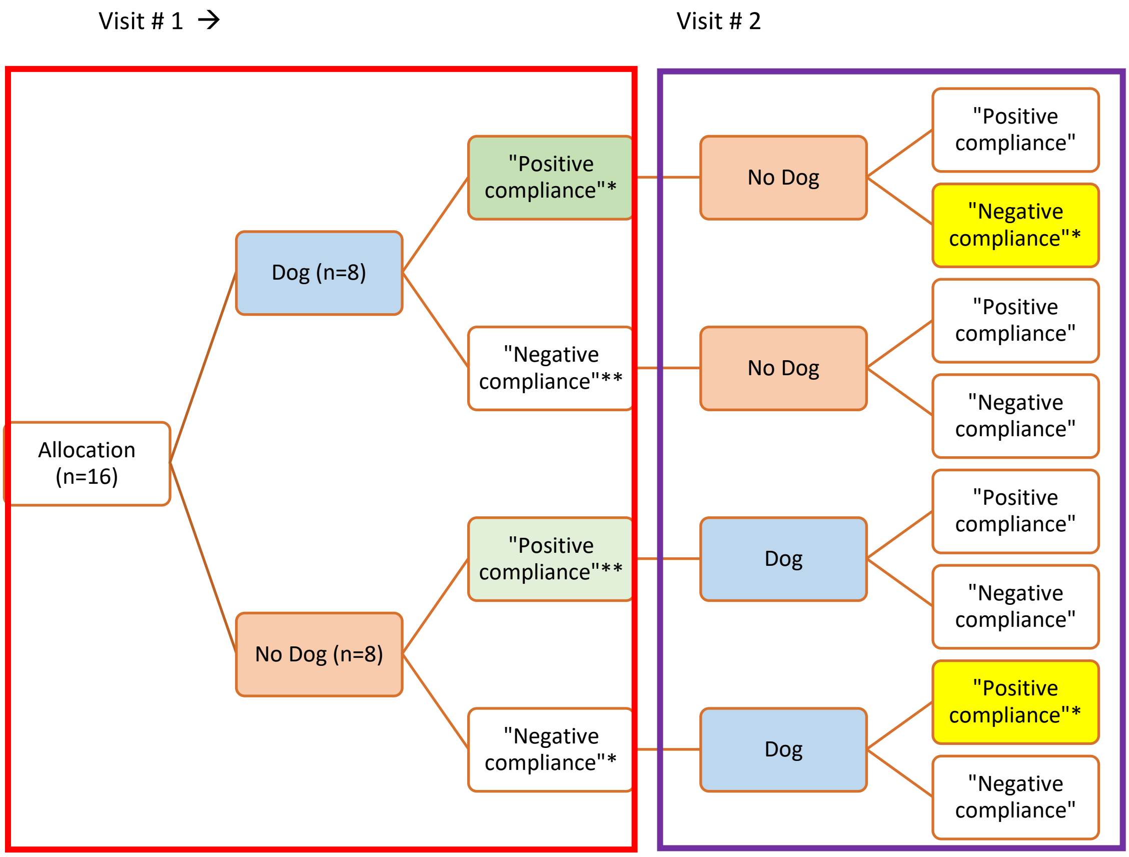

* Expected n= 8, ** Expected n=0

Figure legend:

Study participant flow with anticipated compliance while undergoing an intraoral clinical examination in the presence or absence of a dental therapy dog.
